# Methodological considerations in the evaluation of ventilation feedback devices

**DOI:** 10.1016/j.resplu.2026.101537

**Published:** 2026-10-21

**Authors:** Abdo Khoury, Mohamud Daya, Bernd W. Böttiger, Matthew Neth, Guillaume Debaty, Jean-Baptiste Pretalli

**Affiliations:** a Université Marie et Louis Pasteur, CHU Besançon, Inserm CIC 1431, SINERGIES (UR4662), Department of Emergency Medicine and Critical Care, F-25000 Besançon, France; b Department of Emergency Medicine, Oregon Health and Science University, Mail Code: CDW-EM, 3181 SW Sam Jackson Park Road, Portland, OR 97239, United States; c Institute of Emergency Care, Pedagogy of Health and Nursing Science, University of Pecs, Szigeti str 12, 7624 Pécs, Hungary; d Univ. Grenoble Alpes, Emergency Department and Mobile Intensive Care Unit, CNRS, UMR 5525, Grenoble INP, TIMC, 23 Avenue Maquis du Grésivaudan, 38700 La Tronche Grenoble, France; e Université Marie et Louis Pasteur, CHU Besançon, Inserm CIC 1431, SINERGIES (UR4662), F-25000 Besançon, France

To the Editor,

We read with interest the studies by Orlob et al. and Duchatelet et al. on ventilation feedback devices during cardiopulmonary resuscitation (CPR).^1^, ^2^ Both show that compression-induced reverse airflow can disrupt airflow-based breath detection, producing abnormal ventilation rates and tidal volumes. Because device performance depends on the device itself, as well as on the users and the clinical environment, validation should progressively reproduce the conditions of intended use, as closely as feasible. These findings raise methodological considerations for evaluating ventilation feedback devices, including testing conditions, performance comparability across ventilation feedback devices, and the choice of experimental models.

First, the EOlife® ventilation feedback device is designed to monitor manual ventilation. Orlob et al. used an ICU ventilator with 1.5 s of inflation, a 1.5 s inspiratory hold, and 3 s of expiration. For manual bag ventilation, an inflation time of approximately 1 s is recommended and no inspiratory hold is specified.^3^, ^4^ A longer inspiratory phase exposes each breath to more compression, potentially causing reverse airflow and breath fragmentation. Accordingly, in the manually ventilated animal, inspiratory tidal volume and rate remained within prespecified acceptable limits, whereas expiratory volume did not. This may contribute to discrepancies between ventilation feedback device and reference measurements and should be considered when extrapolating to manual ventilation.

Secondly, parallel evaluation of ventilation feedback devices raises a methodological issue. Devices within the same functional category may identify and measure different metrics. In Duchatelet et al., breath-by-breath pairing was not possible for EOlife X®, whereas it was possible for AccuVent®. Moreover, EOlife X® reports expiratory tidal volume, whereas the reference standard and AccuVent® use an inspiratory metric. Comparing devices that measure different quantities, particularly when ventilation events and tidal-volume metrics are defined differently, may complicate interpretation and comparison of device performance.^4^, ^5^, ^6^

Third, both studies evaluated EOlife X®, a training device intended for use on manikins, rather than EOlife®, the medical device intended for clinical use in adult cardiac arrest.^7^ Manikin respiratory mechanics can vary substantially and affect measured ventilation.^8^, ^9^ The concordance of findings across the manikin and porcine models supports the evidence that this phenomenon can occur under experimental conditions. However, it does not establish that these findings generalize to EOlife® or to patients undergoing cardiac arrest. Moreover, the magnitude and characteristics of compression-induced airflow are expected to depend on the mechanics of the respiratory system and chest wall.^10^ Clinical evaluation during cardiac arrest is needed to establish whether performance observed in experimental models translates to the intended clinical use.

Future studies should, whenever possible, evaluate the device intended for clinical use, reproduce relevant physiological and technical conditions, and clearly define ventilation events and the reference tidal-volume metric. This would help distinguish device performance from differences arising from experimental conditions or measurement definitions.

We thank Orlob et al. and Duchatelet et al. Their work highlights the importance of validating ventilation feedback device accuracy while also illustrating the methodological complexity of defining what is measured, under which conditions, and whether measurements from different devices can be validly compared.

## CRediT authorship contribution statement

**Abdo Khoury:** Writing – review & editing, Conceptualization. **Mohamud Daya:** Writing – review & editing. **Bernd W. Böttiger:** Writing – review & editing. **Matthew Neth:** Writing – review & editing. **Guillaume Debaty:** Writing – review & editing. **Jean-Baptiste Pretalli:** Writing – review & editing, Writing – original draft.

## Declaration of competing interest

Professor Khoury is Medical Advisor for Archeon Medical and is an inventor involved in the development of the EOlife® ventilation feedback device discussed in this letter.

For the remaining authors, there are no conflicts of interest.
